# Supplementary material for: Are carbon emissions trading and green financial instruments synergistic? -Comprehensive quantitative research based on content analysis
Source: PLoS One. 2024 Mar 7;19(3):e0298601. doi: 10.1371/journal.pone.0298601 (PMC10919601; doi:10.1371/journal.pone.0298601)
Supplement: S1 Data — (ZIP) [file pone.0298601.s001.zip › Data/地级市Z_F工作报告.zip!/║╙─╧/╨┬╧τ╩╨/└┤╫╘╣1⁄2╓┌║┼í╛┬φ┐╦╩2╛▌═°í┐_2017.docx]

各位代表：

　　现在，我代表市人民政府，向大会作工作报告，请予审议，并请各位政协委员和列席会议的同志提出意见。

　　一、2016年工作回顾

　　2016年，市人民政府深入贯彻党中央治国理政新理念新思想新战略，在省委、省政府和市委的坚强领导下，在市人大、市政协的监督支持下，牢牢把握稳中求进工作总基调，着力稳增长、调结构、促改革、防风险、惠民生，较好完成了全年目标任务。主要抓了以下工作：

　　（一）全力稳定经济运行。把稳增长作为全局工作的突出任务，综合施策应对，奋力扭转颓势。完成地区生产总值2140.7亿元，增长8.3%，同比提高2.3个百分点；一般公共预算收入148.1亿元，增长4.1%；规模以上工业增加值增长8.5%，同比提高2.7个百分点，高于全省0.5个百分点；工业用电量增速同比提高5.9个百分点。一是扩大有效投资。新开工亿元以上项目245个，同比增加46个。总投资20亿元的中纺院年产10万吨绿色纤维等一批重大项目先后实施。新开工民间投资项目986个，同比增加338个；完成投资783.7亿元，同比增长9.6%，对全市投资增长贡献率达94.9%。二是强化载体支撑。实施产业集聚区建设“五大工程”，产业集聚区规模以上工业增加值增速高于全市水平3个百分点。在全省重点项目暨产业集聚区建设观摩点评活动中，红旗区特色商业区夺得豫北片区第1名，平原示范区夺得豫北片区城乡一体化示范区第1名。三是优化金融生态。开展资本运作服务年和金融知识普及年活动，出台加快金融业发展的22条意见。严厉打击非法吸收公众存款犯罪，化解企业担保链风险，155家企业与金融机构签订主办银行合作协议，124家企业建立债权人委员会制度；组织各类银企对接活动30余次，签约金额417.5亿元，实际到位资金178.9亿元；新增新三板挂牌企业16家，总数达30家，数量居全省第3位。

　　（二）着力优化产业结构。落实供给侧结构性改革的各项要求，蓄积发展新动能。一是谋划工业提质发展。围绕《中国制造2025》及河南行动纲要，结合新乡实际，明确大企业（集团）培育、先进制造业专业园区建设两大抓手，出台政策文件，强化工作举措，并落实到具体项目，推进与瑞典山特维克集团、台湾友嘉实业集团、中兵集团、中航工业、北汽集团、北京大学基础医学院、平煤神马集团等企业和科研院所合作，努力打造基地型项目、龙头型企业。同时，化解过剩产能，退出煤炭产能15万吨；淘汰落后产能，列入年度淘汰计划的造纸、水泥等行业的10户企业、20条（台）生产线（设备）全部拆除。二是促进农业提质增效。粮食受自然灾害影响有所减产，但仍处于历史第二高位，总产保持在70亿斤以上。种植结构持续优化，经济作物面积同比增长3.6%。新增省级农业产业化集群4个、龙头企业13家。三是推动服务业提速发展。服务业增加值增速连续8个季度高于第二产业增速，对经济增长贡献率达46.9%，提高10.4个百分点。电子商务、云计算、大数据等新业态、新模式不断涌现，软通动力华中地区产业互联网总部落户我市，新乡大数据产业园华为云计算数据中心开工建设。接待国内游客人数和旅游综合收入分别同比增长12.6%和 15.1%。

　　（三）全面深化改革开放。坚持激发内力与借助外力相结合，发展活力动力不断增强。一是深化重点领域改革。坚持全面发力、重点突破，年初确定的219项重点改革任务顺利推进。行政审批制度改革方面，精简行政审批事项15项，清理规范中介服务122项；市县两级全面实行权力清单、责任清单制度，编制公共服务事项清单并向社会公开。国企改革方面，从制度规范、机构整合、运作模式等方面重新定位国资监管，纳入改制范围的54户市属国有工业企业完成改制49户。投融资体制改革方面，加快财政性涉企资金基金化改革，注册基金7支，总规模42.3亿元。63个总投资618.5亿元的项目纳入省PPP项目储备库，数量居全省第3位。商事制度改革方面，全面实施企业登记“五证合一”、个体工商户“两证整合”，新登记各类市场主体4.3万余户，同比增长16.2%。农村改革方面，全国农村改革试验区试验成果转化数量在全国58个试验区中位居第2位。土地承包经营权确权登记颁证进展顺利，承包合同签订基本完成，证书颁发工作全面启动。医药卫生体制、公安管理体制、交通运输执法体制等领域的改革取得新进展。二是扩大对外开放。大力开展“一招四引”，瞄准国际、国内领军企业，承接发达地区产业转移。全年新签约亿元以上项目169个，总投资787亿元；实际利用外资增长7.5%，实际到位省外资金增长8%。举办招商引智活动9次，签约合作项目46项，引进或共建研发机构9个。

　　（四）大力开展污染治理。加强生态环境保护，狠抓重点领域治理，环境质量有所改善。一是全力打好大气污染防治攻坚战。强化“六控”措施落实，全面打响“五项围城”清理整顿、“小、散、乱、差”企业整治、燃煤小锅炉拆改三大战役。全年排查出的1691个“五项围城”问题全部整治到位；取缔清退、关停淘汰“小、散、乱、差”企业454家；取缔10蒸吨以下燃煤小锅炉1015台。完成省政府下达的黄标车和老旧车淘汰任务，打掉黑加油站点548个，夏收、秋收期间秸秆焚烧火点数同比减少66.7%。全年PM10、PM2.5平均浓度分别下降8.9%、10.6%，优良天数增加14天，空气质量综合指数退出全国后10名，在全省排名同比提升5个位次，扭转了主要污染物指标不降反升的被动局面。二是健全工作机制。在全省率先出台《党委、政府及有关部门环境保护工作职责》，强化“党政同责、一岗双责、失职追责”，建立完善环境保护“责任体系、监测监控体系、考核体系”三个体系，“排查治理、跟踪监督、奖惩激励”三个机制，实施最严格的督导考核、最严厉的问责追究。探索“专家引路、精准治污”，聘请高水平专家团队开展技术指导，加强污染天气应急管控，精准治污水平进一步提高。三是统筹推进生态文明建设。编制水生态文明建设规划，加快实施中心城区水系连通工程，定国湖基础建设完成工程量的70%，启动16座雨污水泵站改造工程，市区60个排污口全部整治到位，环保部解除对我市涉水项目环评限批。新增造林19.8万亩，基本完成南水北调中线干渠绿化。

　　（五）持续保障改善民生。全年各项民生支出247亿元，同比增长6.3%，占全市财政支出的76%。城镇居民和农村居民人均可支配收入分别同比增长6.1%和7.7%。一是推进脱贫攻坚。坚持精准扶贫，制定出台“1+16”精准扶贫政策体系，突出抓好产业扶贫、易地扶贫搬迁等重点难点工作，90个贫困村退出贫困序列，3.53万农村贫困人口实现稳定脱贫。黄河滩区居民迁建第一批试点5个村基本搬迁安置完毕，第二批试点实施方案获得省政府批复，争取上级资金4.1亿元。二是做好抗洪抢险。面对“7·9”“7·19”两场特大暴雨洪涝灾害，周密部署、科学应对，万众一心、众志成城，组织迁安转移群众6.2万余人，实现了“确保人民群众生命安全，确保主要河道堤防和水库安全，确保国家重点工程和交通要道安全”的目标。三是开展“双城”创建。着力清除脏乱差等城市管理顽疾，城市管理水平、市民文明素质进一步提升，顺利通过国家卫生城市届满复审；全国文明城市创建取得阶段性成效，在全国123个同类城市中，取得了第4名的好成绩。四是统筹做好各项民生工作。城镇新增就业10.5万人，农村劳动力转移就业124.9万人。扩充义务教育资源，市区新增学位6210个，各县（市）新增学位2.8万个；抓好学前教育，新建、改扩建幼儿园257所；“全面改薄”超额完成省定任务，设施设备类项目完成率102%；本科上线率55.09%，高于全省4.6个百分点；落实资助资金7400万元，惠及9万名贫困学生；接收8386名进城务工人员随迁子女入学，连续14年做到“应入尽入、同城同待遇”。强化社会保障，两次提高城乡低保、五保保障标准，农村居民年人均最低生活保障标准由2600元提高到3030元，农村特困人员集中供养标准由每人每年3800元提高到5000元；城市低保月人均补助水平由225元提高到260元，农村低保月人均补助水平由115元提高到150元。加强公共卫生服务体系建设，新农合参保率99.68%，为群众减少医疗费用支出9500余万元。解决2.44万农村居民的饮水安全问题。市区供热普及率达63.2%，同比提高10.2个百分点。深入开展平安建设，生产安全事故起数和死亡人数实现“双下降”，食品药品安全监管全面加强。集中化解信访积案，维护群众合法权益，越级访、非法访数量明显下降。强化社会治安综合治理，严厉打击各类违法犯罪活动，群众安全感、满意度持续提升。国防动员和双拥共建深入开展，蝉联全国双拥模范城“六连冠”。

　　（六）推动重大战略实施。围绕国家、省重大战略布局，谋划推动打基础、利长远的重大事项。一是实施重大战略。郑洛新国家自主创新示范区成功获批，成为在我市重点实施的第一个真正意义上的国家级战略。我们借鉴先进地区经验，突出自身特色，明确“一区四园”发展架构，完善“N+1”政策体系，研究出台新乡片区实施方案和意见，发展规划编制工作稳步推进。首期10亿元的自创区发展基金正在加快推进。二是优化空间布局。对接全省建设组合型大都市地区战略部署，谋划推动郑新产业带、沿黄经济带、南太行文旅康养产业带和国家自主创新示范区（新乡片区）、先进制造业集聚集群创新发展示范区、综合保税区“三带三区”战略布局。共实施平原示范区印刷科技产业园一期、原阳县金祥家具产业园等150个郑新融合项目，完成投资110亿元。大东区区域协同发展战略规划通过专家组评审，建立总投资1753亿元的3年滚动项目库。启动沿黄经济带规划编制。南太行旅游品牌的知名度进一步提升。三是推动重大工程。成功争取郑济铁路济南方向从新乡东站引出，并在我市设立3个站点；成功争取郑济高铁黄河特大桥“一桥四线、公铁两用”；成功争取郑济铁路设计施工时，在高铁新乡东站预留新焦城际铁路接入条件，为郑济、郑太铁路跨线运行打下基础。新晋高速、鹤辉高速、107国道东移改线等重大工程顺利推进。平原路东延等7个新建工程及劳动桥等7个改扩建工程全部竣工。列入省定台账的7个棚户区（城中村）改造项目全部开工。

　　我们以深入开展“两学一做”学习教育为契机，不断加强政府自身建设。全面推进依法行政、从严治政，认真执行市人大及其常委会各项决议决定，自觉接受市人大法律监督和工作监督、市政协民主监督、社会监督、舆论监督，认真办理人大代表建议和政协提案，支持民主党派、工商联和无党派人士参政议政。加强党风廉政建设，严格落实中央八项规定精神，驰而不息整治“四风”，大幅压缩“三公”经费支出，强化政府投资项目和财政资金监管，规范公共资源配置交易，促进了廉洁政府建设。民族宗教、人民防空、统计审计、新闻出版、外事侨务、史志档案、气象地震等工作都取得新成绩。

　　各位代表！

　　一年来，面对各种困难，全市上下团结一心、负重前行，取得了来之不易的成果。这是省委、省政府和市委正确领导的结果，是市人大、市政协监督支持的结果，是全市人民共同努力的结果，凝聚着社会各界的聪明才智、心血汗水，饱含了驻新单位的大力支持。在此，我谨代表市人民政府，向所有参与、支持、关心新乡发展的同志们、朋友们，表示衷心的感谢并致以崇高的敬意！

　　我们也清醒认识到，发展中还存在不少困难和问题。一是经济下行压力较大。部分经济指标增速下滑，一般公共预算收入、固定资产投资增速低于预期；经济运行的质量和效益偏低；部分行业产能过剩，一些企业运营困难，经济稳中向好的基础还不稳固。二是转型升级任务艰巨。供给侧结构性改革推进不快；工业结构层次偏低，三次产业发展不够协调，城乡二元结构依然明显，县域综合实力较弱，自主创新能力有待提高，经济增长的内生动力依然不足。三是环境约束趋紧。资金、土地等资源要素瓶颈制约较为明显，特别是环保形势依然严峻，粗放生产方式造成的生态环境风险日益凸显。四是民生领域欠账较多。基本公共服务均等化程度较低，民生投入刚性需求较大，医疗、教育等方面的短板依然突出，群众对一些领域还有不满意的地方。五是自身建设仍需加强。简政放权还不到位，个别职能部门工作效率不高，一些干部不敢为、不愿为、不会为的问题比较突出，少数干部以权谋私、贪污腐败的问题依然存在。对此，我们一定坚持问题导向，采取有力措施加以解决。

　　二、2017年工作安排

　　在全球经济增长乏力、国内经济调整转型的背景下，新乡既面临着提质发展、转型发展的艰巨任务，又面临着保护环境、推动绿色发展的千钧重担。但我们更要看到，一方面，国家已经确立适应经济发展新常态、以新发展理念为指导、以供给侧结构性改革为主线的政策架构，经济政策更稳、更准、更活、更实、更托底，也更具操作性；另一方面，随着我市在“米”字形高铁布局中的战略意图得到全面体现、郑新融合正式上升为全省战略以及郑洛新国家自主创新示范区加快建设、“三带三区”战略布局逐步展开，将进一步提升新乡战略优势，我们完全能够在打好“四张牌”中作出新乡的贡献、打造新乡的亮点。我们要把思想和行动统一到中央、省委、市委的决策部署上来，既要有顺势借势的智慧，还要有驭势落子的本领，主动融入全国全省大局，突出新乡特色，审时度势，借势发力，努力开创各项事业发展的新局面。

　　今年政府工作总体思路是：全面贯彻党的十八大和十八届三中、四中、五中、六中全会精神，落实中央、省委经济工作会议和市十一次党代会各项部署，统筹推进“五位一体”总体布局和协调推进“四个全面”战略布局，坚持稳中求进工作总基调，牢固树立和贯彻落实新发展理念，适应把握引领经济发展新常态，坚持以提高质量和效益为中心，以推进供给侧结构性改革为主线，着力稳增长、植优势、增活力、补短板、保民生、防风险，促进经济平稳健康发展和社会和谐稳定，推进“四个新乡”建设，加快全面建成小康社会步伐，以优异成绩迎接党的十九大胜利召开。

　　经济社会发展主要预期目标是：地区生产总值增长8%左右、在实际工作中争取更好结果，三次产业比重达到11∶49∶40，一般公共预算收入增长7.5%左右，全社会固定资产投资增长12%，社会消费品零售总额增长11%，城镇化率达到51.6%，城镇登记失业率控制在4.5%以内，居民人均可支配收入与地区生产总值同步增长，节能减排、环境保护等约束性指标完成省定目标。

　　实现上述目标，要按照省委、省政府打好“四张牌”、打赢“四大攻坚战”、做到“三个确保”以及市委抓好“三件大事”的各项决策部署，突出“把经济搞上去、把污染降下来、把风险防控住、把基础打扎实、把民生保障好”五大任务，认真抓好各项工作落实。

　　（一）把经济搞上去。不仅要着力促进经济稳定增长，更要提高发展的质量和效益。

稳定经济运行。加强投资、用电量、税收、利润等指标的监测预测，强化精准调度，提高稳增长措施的有效性。深入开展产销、银企、用工、产学研“四项对接”，推动上下游企业间、政银企间、校企间的深度合作，帮助企业解决难题。强化要素保障，优先保障重大工程、重点项目和民生工程的用地；帮助企业争取各类政策性资金和基金，引导金融机构加大对中小企业的信贷投放，破解融资难题。

　　强化项目建设。落实重大项目市级领导分包、重大项目例会、重点项目专案专班推进、定期观摩、集中开工等工作机制，加快实施一批“十三五”重大项目，特别是“米”字形高铁、107国道东移改线等重大基础设施，城乡电网建设、赵固低热值煤发电等重大能源设施，生态水系等重大民生工程。全年实施省、市重点项目138个，完成投资240亿元以上；力争新乡化纤超细旦氨纶项目一期等163个亿元以上项目竣工投产，中兴通讯大健康产业园、山特维克智能制造产业园等180个亿元以上项目开工建设，亿元以上项目完成投资603亿元。

　　加快转型升级。按照全省转型发展攻坚战的要求，结合新乡实际，把产业结构优化升级与优化城乡结构特别是壮大县域经济结合起来，强化大企业担当、大项目支撑，迅速提升区域整体竞争力。一是壮大县域经济。完善壮大县域经济的政策措施，积极探索项目寄养、托管等方式，完善成本分摊和利益分配机制，引导各县（市）、区合力推进重大项目的引进和实施。鼓励有条件的县（市）、区率先崛起，将政府可控资源向发展态势好、速度快、质量高、干劲大的县（市）、区倾斜，让好马吃饱、好马快跑。引导各县（市）、区深入分析比较优势和短板不足，精准制定各项发展举措，解决制约发展的突出矛盾和关键问题，力争在新一轮区域经济竞争中实现赶超。二是加快产业集聚区建设。继续实施产业集聚区建设“五大工程”，打造一批主导产业突出、关联度高、集聚效应明显的产业基地。力争装备制造产业规模以上工业企业主营业务收入达到1200亿元，食品产业达到480亿元，电池电动车产业达到280亿元，生物新医药产业达到250亿元，各县（市）、区共培育百亿级特色产业集群10个。三是推动先进制造业专业园区建设。落实《中国制造2025河南行动纲要》，争创“中国制造2025”示范城市和河南省先进装备制造示范区。本着研发平台、孵化器、加速器、产业园区，即一台、两器、一区的“121”发展模式，制定建设标准，实行动态管理，鼓励各县（市）、区培育一批“区中园”“精品店”，努力将首批推动的10个专业园区建设成为产业链、价值链、供应链、创新链系统集成的载体平台，打造成为郑洛新国家自主创新示范区（新乡片区）的主要辐射载体，引领产业转型升级。同时，抓好河南省（新乡）军民融合产业基地建设，以“一个基地、三个园区”和中国电波城建设为载体，加大资源整合力度，建设军民融合创新发展示范区。四是强力培育大企业（集团）。重点支持先期筛选的新航集团、科隆集团、白鹭集团、豫飞重工、华兰生物、心连心化肥、克明面业等20家企业发展，引导企业依托先进技术、优势产品、客户资源、销售渠道等比较优势，深化股权改革，引入战略投资者，有效利用资本市场。鼓励我市企业家弘扬企业家精神，建立完善创新、金融、品牌战略，优化企业治理结构，迅速做大做强。我们将努力通过企业组织结构的调整，加快形成大企业“顶天立地”、小企业“铺天盖地”、高新技术企业抢占“发展高地”的新格局。

　　加快新型城镇化。新型城镇化既是扩大内需的最大潜力，也是提升综合竞争力的重要途径。一是积极融入中原城市群发展规划。坚持规划引领，促进“多规合一”，对接中原城市群发展规划，尽快启动城市总体规划修编；完善各类专项规划，高标准启动“三带三区”发展规划、郑新融合全域发展规划、综合交通发展战略规划的编制工作。重点按照“理念引领、交通先行、产业协同、功能互补、节点突破、统筹推进”的思路，加快推进郑新融合发展。一方面，加快郑新基础设施市政化融合，谋划建设向南连接郑州的高速大外环，加快郑新快速通道建设，打造郑新融合的大道、产业发展的大道，为构建郑新产业带提供基础设施支撑。另一方面，发挥平原示范区、原阳产业集聚区、获嘉亢村产业集聚区、新乡经济技术产业集聚区的战略支点作用，进一步强化与郑州在基础设施、产业布局、社会事业、环境保护等方面的对接。二是提升中心城区综合承载力。围绕打造全国一流协同发展示范区的目标，加快推进大东区建设，重点抓好市商务中心区建设，与高铁站枢纽经济区建设一同谋划，做好107国道东移、中心医院东区医院、河南科技学院东校区、大沙河综合治理等项目建设，切实提升公共服务功能。统筹推进铁西区域开发建设，促进新老城区有机更新、均衡发展。以改善群众居住环境为导向，抓好科隆大道东延等112个市政公用基础设施项目建设，重点推进卫滨区铁道新苑二期等3个棚户区和红旗区马小营二期等3个城中村项目，启动劳动南街区域等3个棚户区和东牧村等5个城中村改造项目，基本建成列入省台账的公村等4个棚户区（城中村）改造项目。三是更加注重以城带乡。强力推进百城建设提质工程，高水平编制县（市）城乡总体规划，做好提高综合承载能力、推进产城融合发展、提高人居环境质量、提高精细化管理水平等重点工作，通过县城的改造提质来辐射带动乡村发展。今年，在辉县市、卫辉市纳入全省第一批试点的基础上，其余5个县争取进入第二批试点。同时，高标准谋划建设一批各具特色、富有活力的先进制造、商贸物流、教育科技、文化旅游等特色小镇。

　　提升质量效益。加快推进供给侧结构性改革，推动三次产业提质增效、融合发展，促进生产从粗放向集约、产品从中低端向高中端转变。农业方面，推进农业供给侧结构性改革，围绕国家农业科技园区建设，提升农业经营的专业化、市场化、集约化水平；积极应对国家粮食收储政策调整变化，确保生产能力不降低，加快农业结构调整，优化农业资源配置，增强农产品有效供给，促进农业增效、农民增收。新增高标准粮田20万亩以上、年销售收入10亿元以上的农业产业化集群2个。工业方面，按照“立支柱、举龙头、铸品牌、强载体”的思路，瞄准产业链的终端、价值链的高端、创新链的前端，改造提升传统产业，发展壮大支柱产业，大力发展新兴产业，推动产业发展由跟跑、并行向领跑转变。做好僵尸企业市场出清、化解过剩产能等工作，促进工业强身健体。深入实施质量强市战略，争创全国质量强市示范城市，大力弘扬“工匠精神”，引导企业围绕中高端产品需求，加快新产品开发，加强标准体系建设、品牌培育和质量提升，提高产品层次和水平。服务业方面，重点抓好现代物流、电子商务、金融保险、信息服务、文化旅游、健康养老、现代商贸等主导产业发展。推动“大旅游、大健康、大文化”融合发展，加快全域旅游的规划和开发，以南太行健康旅游文化产业园为龙头，先行先试、探索经验，推动旅游业发展从景区打造向区域产业集群转变；构建“一个中心、三个特色产业园区”的大健康产业体系，打造河南乃至全国的养老健康产业发展示范基地；挖掘商周文化、三国魏晋文化、宋文化、太行文化、红色文化内涵，精心打造具有新乡特色的文化旅游产品，建设全国红色旅游经典景区，真正把文化资源转化为经济资源、发展实力、竞争能力。

　　（二）把污染降下来。牢固树立保护生态环境就是保护生产力、改善生态环境就是发展生产力的理念，落实好实践证明行之有效的机制和举措，坚持做到科学施策、标本兼治、铁腕治污。

　　打好污染防治攻坚战。推动工作思路由总量管理、容量管理向环境质量管理转变，重点算好污染点源账、治理重点账、调控对象账“三本账”，加强对大气污染的源解析研究，落实好每日会商研判机制，全面推行网格化管理，深入推进依法治污、科学治污、精准治污，确保PM10、PM2.5浓度稳定下降，空气优良天数达到189天。打响水污染防治攻坚战，以《新乡市水生态文明建设规划》为引领，按照“规划先行、控源截污、达标治理、清淤疏浚、开源引水”的思路，全面落实“河长制”，一河一策、水陆统筹，系统推进水污染防治、水生态保护和水资源管理，确保5条出境河流水质达到省定标准，集中式饮用水水源水质达到或优于三类比例总体高于96%，城市建成区基本消除黑臭水体。加强水源保障，用足用好黄河水、南水北调水、地下水，推进东大宫等3个引黄口门改造，确保年内建成；启动原阳县、延津县等南水北调配套引水工程建设，为增加区域内生态径流量、改善河流水质提供保障。

　　强化生态文明建设。开展全民、全域绿化行动，完成造林20万亩，努力打造“村在林中、路在绿中、房在园中、人在景中”的点、线、面相结合的城乡绿化体系。谋划建设国家级森林公园，对凤泉湖、凤凰山及周边矿坑区近50平方公里区域进行统筹开发，打造山水林田湖一体化生态城。探索国有林场改革新路径，延津县国有林场要先行先试，高水平编制规划，优化土地资源配置和利用，打造集休闲、文化、体育、旅游于一体的主题森林公园。发挥好滩区生态风景、湿地水文化优势，谋划实施沿黄带状生态公园，按照“成熟一段、建成一段”的原则，打造一批沿黄经济带上的“生态珍珠”。持之以恒、坚持不懈，强力推进以“5湖4河2渠”为重点的水系项目建设，全面完成赵定河综合治理、定国湖项目建设，做好卫源湖、南湖等项目的基础设施建设。

　　提高环境治理水平。完善和落实环境保护“三个体系、三个机制”，统筹考虑自然生态各要素、山上山下、地上地下、流域上下游，着力开展整体保护、系统修复、综合治理，增强生态系统循环能力，切实做到环境治理的系统化、科学化、法治化、精细化、信息化、全民化“六化并举”。只要全社会不懈努力，生态环境就一定能够好起来。

　　（三）把风险防控住。把防控金融风险放到更加重要的位置，高度重视企业经营风险，以及房地产过热可能带来的风险。

　　防控金融风险。加快担保体系重组，推进与河南鑫融基公司的对接合作，通过资产管理、并购基金等多种方式探索化解金融风险。加强地方金融体系建设，加大战略投资者引进力度，推动组建市级农商行，壮大地方金融机构实力。继续落实主办银行制度，充分发挥债权人委员会作用，支持银行化解不良贷款，有效防范和化解企业互保风险；加快非法集资案件司法处置进程，对非法集资保持高压态势，做到露头就打，坚决防止新的案件发生。

　　防控企业经营风险。引导企业强化风险意识，密切注意与企业经营相关的市场、制度、财务、债务等因素的变化趋势，科学监测、预测、预警经营风险；发挥政府引导作用，推动金融机构对有市场、有技术、有效益、有信用、创新能力强的企业，做到不压贷、不抽贷、不断贷，帮助企业渡过难关。探索建立综合性金融服务体系，促进上市挂牌、担保、融资租赁、资产管理、产权基金等多种金融工具形成组合拳，帮助中小企业解决融资难、融资贵问题，化解经营风险。

　　防控房地产风险。坚持住房的居住属性，严禁投机炒房，稳定房地产市场。发挥规划和土地的管控作用，合理控制住房用地供应规模、布局和节奏，促进房地产供需总量平衡和结构优化。强化市场监管，健全开发企业实力和信用评估机制，提高准入门槛，注重引进国内一流房地产开发企业参与新乡城市发展建设。消化商品房库存，切实提高棚户区改造货币化安置比例，鼓励农民工、外来务工人员进城购房。加快老旧住宅小区整治改造，完善配套基础设施建设，提升住宅小区物业管理水平，努力让市民在住有所居中创造新生活。

　　（四）把基础打扎实。以加强重大基础设施建设、促进基础设施互联互通互融为抓手，加强基础能力建设。

　　构建现代综合交通运输体系。加快郑济铁路新乡段及“一桥四线、公铁两用”黄河特大桥建设，推进新焦城际铁路、郑新南铁路前期工作，谋划高铁新乡东站双面站建设。加快新晋高速建设步伐，完成投资10亿元；确保鹤辉高速主线基本贯通；做好林焦高速辉县段、新日高速原阳至封丘段前期工作，争取早日开工。统筹推进新延、新辉、新卫、新获、新原等8条城际快速通道的规划建设工作，推动形成中心城区与各县（市）、各开发区、平原示范区互联互通的“环形+扇形”城市组团交通网；重点推进新延快速通道建设，为推动区域融合发展提供示范。按照“军民融合、平战结合”的原则，推动建设军民两用机场。

　　完善提升科学发展载体。继续深化产业集聚区体制改革，重点抓好先进制造业专业园区发展，提高综合竞争力、带动力。加快服务业“两区”建设，培育“互联网+”、大数据、文化创意、服务外包、专业交易市场等专业园区，力争在建项目达40个以上，完成投资40亿元以上。

　　加快信息基础设施建设。推进“宽带中国”示范城市建设，依托阿里巴巴、谷歌等跨境电商平台，培育发展电子商务、现代物流、移动互联网服务等新业态，建设一体化综合跨境贸易示范园区；依托华为、软通动力等龙头企业，发展大数据、云计算、产业互联网等高端IT服务业，推动智慧城市建设。加快组建大数据交易中心，推动城镇化、工业化、信息化深度融合，打造全省大数据、云计算产业基地。

　　（五）把民生保障好。加大民生投入，办好民生实事，促进公平正义，不断增强人民群众的获得感。

　　深入推进脱贫攻坚。切实把抓好脱贫攻坚作为头等大事和第一民生工程，坚持问题导向和目标导向，勇于克难攻坚，全力以赴打赢脱贫攻坚战。聚焦精准扶贫、精准脱贫，牢牢把握“六个关键环节”，确保贫困户识别零差错、贫困户退出零差错、扶贫资金使用零差错。落实“1+16”政策体系，用好“转、扶、搬、保、救、教”六条路径，切实发挥好产业扶贫的造血功能，统筹做好非贫困县、非贫困村贫困人口脱贫工作，确保再实现4.27万农村贫困人口稳定脱贫。抓好滩区居民迁建第一批试点收尾工作，压茬推进第二批试点，力争更多滩区群众纳入试点范围。

　　千方百计扩大就业。把产业结构调整与扩大就业容量有机结合，围绕承接产业转移和服务业发展开发就业岗位，推进“大众创业、万众创新”，解决好返乡农民工、高校毕业生、退伍军人等群体的就业创业问题，城镇新增就业7.5万人。

　　优先发展教育事业。强力推进“全面改薄”和扩充城镇义务教育资源工程，落实县级政府主体责任，加快义务教育学校建设，完成义务教育发展基本均衡县创建年度任务；合理布局、科学推进优质教育资源合作办校，发挥帮扶带动作用，促进教育公平、均衡发展；保障进城务工人员随迁子女入学。落实好学前教育三年行动计划，再创建1所省级示范幼儿园、5所市级示范幼儿园。积极与清华大学、沪江网开展在线教育合作，加快基础教育信息化建设。发展职业教育，深入挖掘驻新高校优势，加强校地合作，促进地方经济社会发展，抓好河南科技学院新科学院新校区建设。扩大教育对外开放，加快西安交通大学与新乡学院合作建设3D打印学院的步伐，推动北京大学附中在新乡建设分校。

　　完善社会保障体系。健全“五险”统一征缴模式，实施全民参保登记计划，扩大社会保险覆盖面，做好机关事业单位和城镇企业职工退休人员基本养老金待遇调整工作，确保城乡医保信息系统互联互通。全面加快健康新乡建设，把人民健康放在优先发展的战略地位，加强重大疾病防控和儿童、妇女、老年人、残疾人等重点人群健康服务，为需要抢救性康复的530名符合条件的残疾儿童提供救助；统筹做好医药卫生体制改革、城市公立医院改革、分级诊疗和家庭医生签约服务等工作，推进医疗联合体建设，建立现代医院管理制度，加强医疗卫生行业综合监督，不断完善卫生健康服务体系。全市二级以上医疗单位为贫困患者减免诊疗费、住院费等1000万元以上。推进百泉中药材产业基地建设。加快中心医院东区医院、市健康管理与康复中心以及结核病防治所、职业病防治所、妇幼保健院整体搬迁等项目建设，促进医疗卫生与文化、体育、旅游、养生、养老等领域融合发展。

　　维护社会大局稳定。严格落实安全生产责任制，深化重点行业、重点领域专项整治，加强风险管控和隐患排查治理，严防重特大事故发生。食品药品安全事关人民健康，必须管得严而又严。要坚持源头控制、全程严管、重典治乱，加强从田间到餐桌的全过程食品安全监管，强化进口食品检验检疫，严厉打击药品违法行为，坚决把好人民群众饮食用药安全的每一道关口。完善信访工作机制，加强矛盾纠纷调处化解，做好重大项目社会稳定风险评估，及时预防化解各类群体性事件。完善立体化社会治安防控体系，加快推进治安三期视频监控系统建设，深入实施“一村一警”工程，严厉打击各类违法犯罪活动，提高人民群众安全感和满意度。落实防汛抗旱责任，提高灾害性天气监测、预报、预警水平，时刻做好防大汛、抗大灾、抢大险的准备，确保人民群众生命财产安全。

　　以创建全国文明城市为抓手，统筹各项事业发展。坚持创建为民、创建靠民、创建惠民，强力推进标准化、常态化、制度化建设，以解决好市民最关心、最直接、最现实的问题和城市建设管理中最薄弱的环节为重点，围绕测评体系、创建标准、加分项目，在补齐短板、打造亮点上下功夫，确保全国文明城市创建工作决战决胜。大力发展文化、体育事业，抓好平原体育综合训练中心建设，确保平原文化艺术中心投入使用，完成市科技馆布展，加快向全民免费开放公共文化体育设施进程，提升公共文化体育产品供给水平，加快文化产业发展，更好地满足人民群众的精神文化需求。新建或改造市区内公厕190座。完善市区户籍65周岁及以上老年人免费乘坐市内公交车的管理办法，方便市民出行。大力开展温暖新乡建设，修编供热专项规划，完善东区热网，构建老城区大支管格局，力争市区供热普及率达到85%以上。落实好党的民族、宗教和侨务政策。深入开展双拥共建，加强国防教育和国防后备力量建设，支持国防和军队改革，支持驻新解放军、武警部队、预备役部队、民兵和人民防空建设，推进军民融合深度发展。支持群团组织更好发挥桥梁纽带作用。做好统计审计、气象地震、史志档案、新闻出版等工作，促进各项事业全面进步。

　　三、全力保障工作落实

　　适应把握引领经济发展新常态，关键是实现动能转换。我们将进一步加强政府自身建设，不断增强改革、开放和创新三大动力，推动经济持续健康发展。

　　（一）靠改革激发活力。围绕经济增速放缓、传统动力弱化、企业活力不足、资源环境约束趋紧等现实问题，精准发力、重点攻坚，不断提高改革整体效能。强化国企改革攻坚，强力推进市属国有工业企业改革，按照“成熟一户、改制一户”的原则，积极推进局（委）属企业改制，妥善解决历史遗留问题；加快推进剥离国有企业办社会职能工作，做好“三供一业”分离移交、国有企业退休人员社会化管理等工作。深化行政审批制度改革，坚持简政放权、放管结合、优化服务“三管齐下”，年内各县（市）、区全面实施“两集中、两到位”，推行“一个窗口对外、一个机构履职、一次受理到位”，继续清理规范中介机构，降低制度性交易成本。深化投融资体制改革，发挥政府投融资平台作用，完善法人治理结构，加快市场化转型，不断提高对全市经济发展的战略支撑能力。继续推动财政性涉企资金基金化改革，出台政府引导基金管理办法，积极发展产业基金，扶持培育龙头企业、重点行业和新兴产业发展。推进多元融资，推广PPP项目，全面提升县级投融资平台的融资能力。深化农村产权制度改革，加快农村土地承包经营权、农村宅基地和集体建设用地使用权确权登记颁证工作。年内完成第三批农村集体产权股份合作制改革试点任务。建立完善市、县、乡三级农村综合产权交易平台，促进农村各项产权规范交易流转。统筹做好其他领域的改革，在医疗、住房、环保等领域，着力推出符合实际、群众认可的改革实招硬招，建立统一的城乡居民基本医疗保险制度，探索推进租购并举的住房制度，在环境高风险领域实施第三方治理，努力通过改革为群众带来更多实惠。同时，加快电力体制、城市执法体制改革，推进盐业、文化体制等各项改革。

　　（二）靠开放增强实力。坚持以大招商推进大开放，以大开放促进大发展，以开放倒逼体制改革、倒逼结构调整、倒逼转型发展。大力开展“一招四引”，瞄准新乡发展急需的资金、资本、资产、资源，紧盯全球、国内500强企业、知名高校和科研院所，围绕装备制造、动力电池、新能源汽车、电子信息、3D打印、生物医药、节能环保、航空航天等先进制造业和战略性新兴产业，持续加大承接产业转移力度，各县（市）、区至少引进1家投资10亿元以上的重大产业项目，全市引进国内外500强或行业龙头企业5家以上。持续放大政策优势，用足用好各类城市名片，完善“N+1”政策体系，树立零点思维，谋长远、算大账，敢于以资源换技术，以产权换资金，以市场换项目，积极开展股权招商、并购招商等新模式。积极融入“一带一路”建设，引导企业加快“走出去”步伐。着力构建载体平台，加大外向型企业引进力度，积极申建综合保税区、保税物流中心、保税仓库，全力争取河南自由贸易区新乡共建区。依托“一区两港”，引进国内外知名机构和企业建立区域性总部、分支机构、研发中心，引进各金融机构建立数据处理中心、卡中心、产品开发中心，建设中原城市群有影响力的企业总部港、金融港。切实提升招商实效，按照“专题、专案、专班、专人、专责”原则，组建招商项目推进领导小组，着力推动瑞典山特维克集团和安徽驰纬集团智能装备制造产业园、软通动力新乡城市创新综合体、北汽集团、平煤神马集团等一批跟踪洽谈项目尽快签约；推动汇智协同创新谷、首弘智能经济产业园等一批重大签约项目尽快开工，确保合同履约率、项目开工率、资金到位率位居全省前列。在推动产业开放招商的同时，坚持依法依规，加快文化、教育、科技、体育等领域的开放合作步伐。

　　（三）靠创新增添动力。以郑洛新国家自主创新示范区（新乡片区）建设为重要抓手，加快实施创新驱动战略，强化体制机制创新，努力让创新活力竞相迸发。构建开放型空间布局，坚持“一核多区”的发展体系和推进机制，以高新区为核心区，平原示范区、国家化学与物理电源产业园、新东产业集聚区、大学科教城为紧密区，以首批确定的10个先进制造业专业园区作为共建区，在空间布局上形成开放体系，建立吸引其他县（市）、区进入的机制，对创新型、先导性产业园区进行托管，同等享受示范区的优惠政策，实现“一顶帽子大家戴”，逐步构建“中心带动、周边协同、梯次建设、联动发展”的新局面。加快“一区四园”建设，选准主攻方向，打造特色和亮点，力争新乡片区取得更多单项冠军。谋划推动高新区的大数据产业园、新能源汽车产业园、航空航天产业园建设；加快平原示范区的装备制造产业园、生物与新医药产业园、电子信息产业园建设；打造新东产业集聚区的3D打印专业园区；推进牧野区的中国电波科技城、化学与物理电源产业园建设；加快实施经开区的“一城两园”建设，即大学科教城与信息通信产业园区、绿色纤维专业园区建设。扩大对外开放合作，本着“不求所在、但求所为，不求所有、但求所用”的原则，对接国内外科研机构、知名高校及龙头企业，采取务实灵活模式共建研发机构、产业技术研究院、院士工作站等研发平台。加快与中国兵器、北汽集团在北京合作的研发机构建设进度，推进与中电科22所、中科院上海硅酸盐研究所、华大基因、同济大学、西安交通大学、世界华人技术经理人协会的合作。鼓励和支持有条件的企业和单位参与国际科技合作项目实施。培育创新文化，营造尊重知识、崇尚创造、追求卓越的浓厚氛围，有效激发全社会尤其是优秀人才的创业创新热情。研究制定引进高层次人才的办法和措施，不惜“千金买宝刀”，敢于为人才定价，以更优惠的政策、更灵活的方式、更优质的服务引进人才、留住人才、用好人才。持续开展企业家提升培训，引导企业以开放的思维创新发展。建立完善容错机制，宽容失败，允许试错，鼓励创新。

　　（四）全面加强政府自身建设。扎实开展“敢担当、转作风、争一流”实践活动，严格按照“一定要与中心工作、重点工作和本职工作相结合；一定要坚持问题导向，以解决突出矛盾为切入点和突破口；一定要坚持以上率下、率先垂范”的要求，加快推进政府职能转变，切实形成“清新简约、脚踏实地、务本责实”的良好政风，确保实现“六个显著”的目标，努力做出让人民群众满意的实绩。做到心中有党。牢固树立“四个意识”，始终在思想上政治上行动上同以习近平同志为核心的党中央保持高度一致。把守纪律、讲规矩摆在更加重要位置，自觉按照党的组织原则和党内政治生活准则办事，不折不扣执行中央、省委和市委决策部署，确保政令畅通、令行禁止。做到心中有民。坚持以人民为中心的发展思想，把维护好、实现好人民群众根本利益作为政府工作的出发点和落脚点，进一步转变工作作风，密切联系群众，深入基层开展调查研究，着力解决实际问题。做到心中有法。自觉接受市人大的法律监督、工作监督和市政协的民主监督，认真听取民主党派、工商联、无党派人士和人民团体的意见建议，主动接受社会公众和新闻舆论监督。全面落实权力清单、责任清单制度，坚决纠正不作为、乱作为，坚决治理懒政怠政行为。扎实做好“七五”普法工作，全力营造尊法、学法、守法、用法的良好法治氛围。做到心中有责。对重点工作拉清单、立专案、建台账、明责任、设时限、定奖惩，做到任务可量化、指标可考核、过程可追溯、责任可追究，做到挂帅出征、挂图作战、挂牌督办。通过建立完善奖罚分明的结果运用机制，把干与不干、干好与干坏区别开来，强化正面激励，为敢于担当的干部担当，为敢于负责的干部负责，营造干事创业的浓厚氛围。做到心中有戒。始终把纪律和规矩挺在前面，严格落实中央八项规定精神，严格执行新修订的两个《准则》和3个《条例》，驰而不息整治“四风”，保持惩治腐败的高压态势。认真落实党风廉政建设主体责任和“一岗双责”，强化权力运行制约监督，切实扎紧制度笼子，做到干部清正、政府清廉、政治清明。

　　各位代表！

　　新乡正处于爬坡过坎、转型发展的关键时期。让我们更加紧密地团结在以习近平同志为核心的党中央周围，在省委、省政府和市委的坚强领导下，团结拼搏，开拓创新，撸起袖子加油干，扑下身子抓落实，为建设“四个新乡”、决胜全面小康作出新的更大的贡献，以优异成绩迎接党的十九大胜利召开！
